# Supplementary material for: A Likelihood Approach for Real-Time Calibration of Stochastic Compartmental Epidemic Models
Source: PLoS Comput Biol. 2017 Jan 17;13(1):e1005257. doi: 10.1371/journal.pcbi.1005257 (PMC5240920; doi:10.1371/journal.pcbi.1005257)
Supplement: S1 File — (TAR.GZ) [file pcbi.1005257.s014.tar.gz › HSPH_Online-SI-Revision/Additions/MSS11/n100-extreme_noisy/n100-extreme_noisy_NEW_table-4.pdf]

1	R0	{2.76052, 16.3913, 0.1}	{2.44577, 11.9005, 0.}	{{2.32891, 10.9956, 0.}, {2.52368, 12.5037, 0.}}	{11.4019, 27.3977, 100.}	inf	10 419.	8588.34	{8075.36, 8999.42}	17.5704	0.0843585	newly	5869.58	6805.47	{6614., 7019.}	15.9447	0.06413	Reff	2.28524	2.06721	{1.97037, 2.13339}	9.54066	ar	92 090.	88 431.4	{86 611., 89 718.}	3.97286
2	R0	{2.18202, 15.1734, 3.}	{1.60532, 7.45768, 0.99}	{{1.58886, 7.29751, 1.}, {1.58886, 7.29751, 1.}}	{26.8204, 50.9201, 67.}	inf	10 970.	5190.01	{4917.14, 5199.97}	52.9957	0.330449	newly	5914.	5625.56	{5387., 5785.}	5.39804	0.0241153	Reff	1.6841	1.34429	{1.33105, 1.33656}	20.5926	ar	84 264.	64 059.7	{62 934., 64 152.}	24.1436
3	R0	{2.57891, 12.5971, 1.}	{2.13394, 8.80416, 0.}	{{1.47488, 4.06661, 0.}, {2.53075, 11.6597, 0.}}	{17.2543, 30.1096, 100.}	inf	8525.	5760.45	{2462.05, 7762.83}	32.4288	0.223174	newly	6571.	6255.34	{5661., 6591.}	5.47892	0.0250934	Reff	2.202	1.87348	{1.34103, 2.19636}	14.9201	ar	90 302.	77 358.5	{56 232., 89 836.}	14.3336
4	R0	{2.5856, 9.28508, 0.1}	{2.77119, 9.44773, 0.01}	{{2.14326, 6.48739, 0.}, {2.99092, 10.3908, 0.}}	{14.8124, 15.0356, 108.}	inf	2564.	2462.53	{1563.59, 3031.42}	16.4599	0.0797999	newly	3492.	3456.91	{2959., 3958.}	9.19502	0.0404637	Reff	2.47933	2.66166	{2.07919, 2.8574}	14.5429	ar	90 312.	91 356.7	{83 151., 94 036.}	4.66521
5	R0	{2.4406, 12.1341, 1.}	{2.31634, 9.83253, 0.}	{{1.47488, 4.06661, 0.}, {2.86001, 13.5542, 0.}}	{17.5526, 23.6492, 100.}	inf	4472.	3756.39	{1319.43, 5099.26}	28.4329	0.175765	newly	3644.	3931.67	{3298., 4494.}	12.0645	0.0495065	Reff	2.2517	2.15125	{1.40962, 2.63423}	16.4869	ar	88 597.	82 016.4	{56 514., 93 056.}	11.2299
6	R0	{2.72155, 15.4094, 2.}	{2.22946, 10.623, 0.01}	{{2.15677, 10.1363, 0.}, {2.44101, 12.0941, 0.}}	{18.9658, 31.5799, 99.5}	inf	8392.	5871.06	{5105.66, 7584.86}	32.044	0.181505	newly	5118.	5237.48	{4838., 6001.}	6.40485	0.0267863	Reff	2.34317	1.97555	{1.92827, 2.11658}	16.3186	ar	91 682.	83 702.9	{83 190., 88 610.}	8.93361
7	R0	{2.63781, 14.3935, 0.1}	{2.726, 16.365, 0.}	{{1.93539, 9.63533, 0.}, {2.96797, 18.4042, 0.}}	{14.6217, 26.6021, 100.}	inf	9079.	10 441.9	{5260.45, 12 202.1}	30.93	0.131372	newly	5875.	5664.33	{4860., 6012.}	4.97004	0.023129	Reff	2.22919	2.23806	{1.70951, 2.40036}	10.6787	ar	91 192.	90 323.6	{77 819., 93 947.}	5.08469
8	R0	{2.82391, 16.104, 1.}	{2.13193, 9.90652, 0.01}	{{2.15677, 10.1363, 0.}, {2.15677, 10.1363, 0.}}	{24.7884, 38.619, 99.}	inf	8948.	5252.03	{5075.57, 5506.44}	41.9583	0.257572	newly	5170.	4995.14	{4788., 5225.}	4.98298	0.0230192	Reff	2.41478	1.90618	{1.9265, 1.93782}	21.2498	ar	92 664.	81 121.8	{83 166., 83 913.}	12.5197
9	R0	{2.80645, 14.6457, 0.1}	{2.66154, 14.6334, 0.}	{{2.27199, 11.6669, 0.}, {2.7499, 15.3124, 0.}}	{5.16347, 7.64108, 100.}	inf	5809.	5342.48	{3837.66, 5777.41}	8.17301	0.0417605	newly	4055.	3584.19	{3226., 3728.}	11.7359	0.0547968	Reff	2.54301	2.42554	{2.11029, 2.50218}	4.61943	ar	92 567.	90 823.7	{85 741., 92 189.}	1.88326
10	R0	{1.86213, 5.4271, 0.1}	{2.49864, 8.94449, 0.02}	{{1.98388, 6.25659, 0.}, {2.80387, 10.6536, 0.}}	{34.1818, 64.8115, 116.}	inf	1977.	3366.6	{2053.46, 3856.79}	70.6653	0.22183	newly	4393.	4502.98	{3853., 4957.}	9.47826	0.0409855	Reff	1.77612	2.35433	{1.90064, 2.64081}	32.5549	ar	75 362.	88 436.6	{79 368., 92 467.}	17.3491
11	R0	{2.27349, 14.6338, 1.}	{1.82072, 8.6585, 1.01}	{{1.67405, 7.28757, 1.}, {2.33995, 14.464, 1.}}	{25.7305, 46.5296, 1.}	inf	10 491.	6574.92	{5339.18, 10 873.8}	45.67	0.258804	newly	6044.	6366.47	{6141., 6682.}	5.44424	0.0228122	Reff	1.80677	1.50805	{1.40876, 1.84813}	21.0711	ar	85 805.	71 166.7	{67 385., 86 922.}	18.8666
12	R0	{2.77261, 14.1894, 0.1}	{2.62616, 15.3469, 0.}	{{2.46794, 14.1192, 0.}, {2.6365, 15.4074, 0.}}	{5.84576, 8.29594, 100.}	inf	6492.	6120.65	{5204.94, 6390.52}	5.98912	0.027773	newly	4508.	3787.74	{3500., 3945.}	15.9774	0.0760378	Reff	2.48165	2.3571	{2.24542, 2.36651}	5.59102	ar	92 508.	90 737.6	{88 919., 91 131.}	2.02809
13	R0	{2.65402, 19.3935, 2.}	{2.81891, 18.2812, 0.}	{{2.81891, 18.2812, 0.}, {2.81891, 18.2812, 0.}}	{6.2127, 5.73517, 100.}	inf	11 250.	12 016.5	{11 845.8, 12 182.7}	6.81371	0.0286006	newly	4926.	5768.59	{5660., 5871.}	17.105	0.0685297	Reff	2.14402	2.2583	{2.25303, 2.26368}	5.33021	ar	91 184.	92 668.1	{92 486., 92 828.}	1.62756
14	R0	{1.75297, 4.45354, 0.1}	{2.45077, 12.2478, 0.}	{{1.93419, 8.56054, 0.}, {2.96947, 15.963, 0.}}	{39.8066, 175.012, 100.}	inf	699.	1641.	{967.06, 2270.04}	134.97	0.350143	newly	1855.	1405.19	{1122., 1707.}	24.5385	0.13089	Reff	1.72278	2.38062	{1.89682, 2.86885}	38.1844	ar	71 263.	87 403.4	{78 273., 93 901.}	22.6491
15	R0	{1.99928, 7.1501, 0.1}	{2.05119, 7.94494, 0.}	{{1.74092, 5.83416, 0.}, {2.64721, 11.9965, 0.}}	{19.6543, 35.4104, 100.}	inf	3389.	3368.68	{2457.46, 5043.24}	31.8656	0.136928	newly	4922.	4452.98	{4255., 4688.}	9.54368	0.0439139	Reff	1.85853	1.90534	{1.6292, 2.43347}	18.6356	ar	79 602.	77 719.	{70 329., 91 165.}	12.1794
16	R0	{2.67398, 10.3134, 1.}	{2.49101, 11.1284, 0.}	{{2.22376, 9.35432, 0.}, {2.86001, 13.5542, 0.}}	{12.9647, 23.7315, 100.}	inf	4133.	3960.29	{3228.31, 5078.51}	22.7765	0.114832	newly	4823.	3754.47	{3196., 4052.}	22.1549	0.110266	Reff	2.49563	2.31745	{2.07952, 2.63851}	12.0687	ar	91 289.	86 949.4	{84 477., 93 131.}	6.45535
17	R0	{2.44634, 16.3226, 0.1}	{2.19975, 12.8783, 0.64}	{{1.77826, 9.06807, 0.}, {2.81891, 18.2812, 1.}}	{20.7407, 29.5013, 612.}	inf	10 530.	8273.1	{5527.49, 12 113.4}	31.4819	0.169811	newly	5419.	5471.3	{5152., 5773.}	4.01919	0.0172489	Reff	1.9738	1.82195	{1.52154, 2.26039}	17.7531	ar	88 710.	81 368.2	{71 995., 92 726.}	11.4073
18	R0	{2.02168, 10.1676, 0.1}	{2.08276, 11.2077, 0.}	{{2.02235, 10.6804, 0.}, {2.02235, 10.6804, 0.}}	{3.02104, 10.2292, 100.}	inf	4597.	5203.93	{4759.8, 5037.16}	13.2071	0.0460248	newly	4153.	4198.09	{4031., 4306.}	2.53528	0.0107098	Reff	1.82331	1.86034	{1.81404, 1.81952}	2.73802	ar	80 299.	81 147.3	{79 886., 80 732.}	1.38076
19	R0	{2.89969, 15.009, 0.1}	{2.38621, 12.3051, 0.}	{{1.93419, 8.56054, 0.}, {2.7499, 15.3124, 0.}}	{17.7082, 20.2387, 100.}	inf	6390.	4820.64	{3667.72, 5802.67}	24.5597	0.131756	newly	4254.	3897.6	{3579., 4322.}	9.04937	0.0421519	Reff	2.60468	2.17655	{1.77565, 2.49931}	16.4369	ar	93 349.	85 675.4	{77 467., 92 163.}	8.22039
20	R0	{2.68933, 7.34928, 0.1}	{2.04264, 11.5408, 0.01}	{{1.35081, 4.06661, 0.}, {2.7499, 18.4042, 0.}}	{26.01, 72.8182, 108.}	inf	478.	160.098	{4.82236, 460.04}	75.6957	0.936081	newly	1059.	231.57	{3., 680.}	79.4646	1.27863	Reff	2.66728	2.03588	{1.3507, 2.74576}	25.7206	ar	91 574.	72 935.4	{45 965., 92 040.}	20.7976
21	R0	{2.15352, 9.28074, 0.1}	{2.28267, 9.53181, 0.}	{{1.74092, 5.83416, 0.}, {2.64721, 11.9965, 0.}}	{22.0406, 33.2148, 100.}	inf	3961.	3933.99	{2413.19, 5103.19}	30.7848	0.148661	newly	4282.	4333.25	{3913., 4609.}	5.28234	0.0233773	Reff	1.99267	2.11121	{1.62895, 2.43557}	21.0572	ar	83 845.	82 419.4	{70 450., 91 200.}	12.0789
22	R0	{2.21953, 9.04387, 0.1}	{2.33251, 8.90692, 0.01}	{{1.53501, 4.02511, 0.}, {2.9884, 12.8326, 0.}}	{26.0618, 37.4081, 108.}	inf	3707.	3502.5	{1417.49, 5237.3}	38.719	0.203872	newly	4322.	4278.71	{3666., 4638.}	6.14785	0.0275448	Reff	2.06581	2.17479	{1.46482, 2.75504}	25.0048	ar	84 856.	80 735.5	{59 884., 94 037.}	14.7663
23	R0	{2.21987, 14.5347, 1.}	{2.33893, 14.3749, 0.01}	{{2.30907, 14.0824, 0.}, {2.48143, 15.7026, 0.}}	{5.36321, 4.31461, 99.}	inf	10 313.	11 301.2	{10 859.5, 12 483.1}	9.58169	0.0387868	newly	5905.	6613.14	{6478., 6752.}	11.9922	0.0491306	Reff	1.76493	1.8273	{1.80711, 1.90172}	3.53359	ar	85 067.	86 820.9	{86 102., 88 835.}	2.06182
24	R0	{2.84268, 17.9659, 1.}	{2.11182, 12.7457, 0.}	{{2.11132, 12.644, 0.}, {2.11292, 12.9719, 0.}}	{25.7103, 29.0563, 100.}	inf	10 277.	7033.47	{6260.08, 7496.73}	31.5611	0.165948	newly	5220.	4779.82	{4185., 5110.}	8.43295	0.0396963	Reff	2.35894	1.80802	{1.78903, 1.84522}	23.3548	ar	92 836.	82 485.8	{82 108., 82 820.}	11.1489
25	R0	{2.52706, 10.3679, 0.1}	{2.75371, 16.6229, 0.}	{{1.93539, 9.63533, 0.}, {2.96797, 18.4042, 0.}}	{14.962, 61.8849, 100.}	inf	4409.	5918.47	{3099.14, 6857.18}	41.0838	0.151898	newly	4655.	3392.82	{2843., 3636.}	27.1145	0.139053	Reff	2.33579	2.48623	{1.81088, 2.66303}	12.8462	ar	89 623.	91 091.5	{78 225., 93 946.}	4.84646
26	R0	{2.62835, 19.1088, 1.}	{1.99087, 10.728, 0.45}	{{1.77826, 9.06807, 0.}, {2.0968, 12.1899, 1.}}	{24.5442, 43.8583, 57.}	inf	12 549.	7545.62	{5626.65, 8525.36}	39.8708	0.226821	newly	5505.	5983.14	{5265., 6485.}	11.0256	0.0450373	Reff	2.05547	1.65541	{1.52272, 1.72016}	19.8618	ar	90 908.	78 931.6	{72 307., 82 123.}	13.2509
27	R0	{2.76831, 19.4155, 2.}	{2.18554, 11.7676, 0.92}	{{1.95791, 9.82151, 0.}, {2.60465, 15.5255, 1.}}	{21.4822, 39.3908, 54.}	inf	16 382.	10 927.1	{8602.75, 14 375.3}	33.8831	0.186206	newly	6900.	7769.66	{7351., 8147.}	12.6038	0.0512135	Reff	1.96727	1.683	{1.56221, 1.90745}	14.795	ar	92 175.	83 328.9	{78 253., 90 420.}	9.70593
28	R0	{2.96609, 16.8903, 1.}	{1.73376, 5.42097, 0.}	{{1.737, 5.43578, 0.}, {1.737, 5.43578, 0.}}	{41.5472, 67.9048, 100.}	inf	11 248.	3893.68	{3678.68, 4096.63}	65.3834	0.46123	newly	6075.	7102.13	{6713., 7394.}	16.9075	0.0674871	Reff	2.44352	1.5615	{1.55863, 1.57014}	36.0966	ar	94 024.	70 481.7	{70 069., 71 174.}	25.0386
29	R0	{2.17046, 14.39, 0.1}	{2.50772, 16.4781, 0.05}	{{2.11132, 12.644, 0.}, {2.56757, 17.005, 0.}}	{16.138, 17.1801, 142.}	inf	8850.	10 624.8	{7583.52, 11 237.1}	23.6007	0.092989	newly	5160.	5532.86	{5122., 5704.}	7.77054	0.03238	Reff	1.78813	2.02921	{1.79402, 2.07053}	13.484	ar	84 001.	89 132.1	{82 773., 90 282.}	6.50739
30	R0	{2.88772, 12.4647, 1.}	{2.85687, 9.76338, 0.02}	{{2.17292, 6.48739, 0.}, {2.99092, 10.3743, 0.}}	{6.527, 21.6718, 98.}	inf	3959.	2794.1	{2479.01, 3064.88}	29.4241	0.157184	newly	3670.	3859.37	{3407., 4092.}	8.94523	0.0360657	Reff	2.71599	2.73163	{2.07955, 2.86122}	7.68011	ar	93 273.	92 376.5	{83 768., 94 129.}	2.12808
31	R0	{2.46831, 8.14308, 0.1}	{2.28244, 6.35048, 0.}	{{1.88643, 4.56746, 0.}, {2.77394, 8.24535, 0.}}	{16.9309, 24.4907, 100.}	inf	1290.	1153.22	{684.38, 1810.75}	35.5627	0.173373	newly	2181.	2658.67	{1601., 3478.}	36.9807	0.144526	Reff	2.41433	2.2337	{1.84976, 2.6981}	16.4827	ar	88 762.	83 863.7	{76 038., 92 364.}	8.47337
32	R0	{1.93236, 7.38987, 0.1}	{1.91529, 6.40742, 0.06}	{{1.53501, 4.02511, 0.}, {2.9884, 12.8326, 0.}}	{24.9435, 43.8406, 148.}	inf	2899.	2490.34	{1383.99, 5121.5}	46.1887	0.233498	newly	4137.	4108.67	{3645., 4587.}	7.29732	0.0319922	Reff	1.80908	1.804	{1.46399, 2.74598}	23.5642	ar	77 654.	71 100.5	{59 916., 93 900.}	19.2225
33	R0	{2.82251, 17.6954, 1.}	{1.4632, 4.14015, 0.02}	{{1.44762, 4.00571, 0.}, {1.44762, 4.00571, 0.}}	{48.1595, 76.6033, 98.}	inf	8313.	2041.09	{1841.36, 2109.45}	75.447	0.616795	newly	4307.	4632.86	{4365., 4947.}	8.0274	0.0331723	Reff	2.44362	1.36049	{1.34457, 1.35043}	44.3245	ar	92 745.	55 295.	{53 950., 55 608.}	40.3796
34	R0	{1.87083, 12.3994, 1.}	{1.25474, 3.26445, 0.}	{{1.25474, 3.26445, 0.}, {1.25474, 3.26445, 0.}}	{32.9314, 73.6725, 100.}	inf	6523.	1791.67	{1675.01, 1924.5}	72.5331	0.56182	newly	4361.	4250.3	{3949., 4532.}	4.7269	0.0210586	Reff	1.57679	1.10776	{1.10477, 1.11067}	29.7462	ar	76 282.	37 569.7	{36 364., 38 783.}	50.749
35	R0	{2.95779, 16.3003, 1.}	{2.38506, 11.4232, 0.}	{{2.32891, 10.9956, 0.}, {2.52368, 12.5037, 0.}}	{19.3794, 29.9203, 100.}	inf	6128.	4138.35	{4000.75, 4556.43}	32.4681	0.191444	newly	3852.	3645.06	{3460., 3862.}	5.86293	0.0276513	Reff	2.67834	2.20758	{2.15211, 2.33494}	17.674	ar	93 873.	85 803.5	{86 468., 89 678.}	8.59617
36	R0	{2.18718, 14.261, 3.}	{1.24751, 2.5364, 0.}	{{1.24751, 2.5364, 0.}, {1.24751, 2.5364, 0.}}	{42.9625, 82.2144, 100.}	inf	9128.	1746.98	{1624.38, 1857.87}	80.8613	0.718662	newly	5307.	5351.36	{4972., 5657.}	4.45902	0.0192562	Reff	1.78461	1.09959	{1.09645, 1.10314}	38.3846	ar	84 379.	36 919.5	{35 496., 38 159.}	56.2456
37	R0	{2.44912, 9.31118, 0.1}	{2.49172, 8.91645, 0.02}	{{1.98388, 6.25659, 0.}, {2.80387, 10.6536, 0.}}	{10.4589, 10.7921, 116.}	inf	3260.	3323.43	{1961.07, 3866.36}	19.822	0.0932228	newly	3995.	4440.59	{3642., 4956.}	14.4268	0.0581265	Reff	2.30876	2.34844	{1.90227, 2.63981}	9.80571	ar	88 442.	88 239.5	{79 232., 92 536.}	4.52355
38	R0	{2.05949, 5.72514, 0.1}	{1.95425, 6.87451, 0.}	{{1.47488, 3.83841, 0.}, {2.9884, 12.8326, 0.}}	{20.594, 37.5303, 100.}	inf	809.	835.528	{331.431, 1813.98}	43.4901	0.191424	newly	2064.	1340.82	{798., 1794.}	35.0378	0.207295	Reff	2.02491	1.91871	{1.45639, 2.90004}	20.2159	ar	81 587.	73 706.6	{56 399., 93 835.}	15.4798
39	R0	{1.75505, 8.52404, 0.1}	{2.02897, 10.4895, 0.02}	{{1.93539, 9.63533, 0.}, {1.93539, 9.84456, 0.}}	{15.6074, 23.058, 116.}	inf	4468.	5702.28	{5114.14, 5547.78}	27.625	0.09672	newly	4543.	4896.39	{4748., 5042.}	8.04292	0.0335268	Reff	1.55847	1.7804	{1.70548, 1.71569}	14.2406	ar	70 925.	79 273.1	{77 323., 78 516.}	11.7703
40	R0	{2.24724, 13.7861, 1.}	{1.24751, 2.5364, 0.}	{{1.24751, 2.5364, 0.}, {1.24751, 2.5364, 0.}}	{44.4869, 81.6017, 100.}	inf	10 101.	1745.48	{1599.45, 1881.94}	82.7197	0.763192	newly	6145.	5362.87	{4870., 5721.}	12.752	0.0601765	Reff	1.78404	1.09976	{1.09649, 1.10299}	38.3555	ar	85 257.	37 017.9	{35 893., 38 127.}	56.5808
41	R0	{1.77635, 8.62406, 0.1}	{2.42626, 12.6563, 0.91}	{{2.32891, 10.9956, 0.}, {2.85582, 17.2484, 2.}}	{36.587, 47.2384, 896.}	inf	5330.	9069.95	{7916.03, 12 380.9}	70.3486	0.221232	newly	5334.	6570.12	{5857., 7075.}	23.1744	0.0892761	Reff	1.53452	2.02129	{1.96651, 2.27286}	31.7216	ar	72 941.	87 520.1	{86 354., 93 030.}	19.9876
42	R0	{1.80397, 5.4932, 1.}	{2.60688, 9.47038, 0.03}	{{2.59708, 9.39342, 0.}, {2.59708, 9.39342, 0.}}	{44.5079, 72.4019, 97.}	inf	2276.	3814.15	{3572.15, 3970.2}	68.1954	0.222937	newly	4341.	4837.87	{4530., 5040.}	12.5324	0.0503568	Reff	1.7106	2.44177	{2.43004, 2.44402}	42.7436	ar	73 232.	90 452.2	{90 272., 90 835.}	23.5146
43	R0	{2.83096, 16.7077, 0.1}	{2.55974, 16.4592, 0.}	{{2.30907, 14.0824, 0.}, {2.93978, 19.9817, 0.}}	{11.9636, 13.8503, 100.}	inf	8628.	8246.56	{6899.63, 10 327.5}	16.5455	0.0740088	newly	4809.	4469.21	{4329., 4651.}	7.06571	0.0325367	Reff	2.43862	2.19117	{2.00458, 2.46008}	10.6442	ar	92 740.	89 459.2	{86 256., 93 677.}	4.13835
44	R0	{2.73678, 11.6021, 1.}	{2.2952, 8.36744, 0.05}	{{2.26582, 8.12562, 0.}, {2.26582, 8.12562, 0.}}	{16.561, 29.0521, 95.}	inf	6552.	5203.87	{4900.98, 5324.83}	21.4733	0.104903	newly	6185.	6794.85	{6557., 7102.}	10.8826	0.0450057	Reff	2.44682	2.07432	{2.04461, 2.05556}	15.6154	ar	92 003.	85 976.1	{85 375., 85 874.}	6.6467
45	R0	{2.14235, 6.98601, 0.1}	{2.52764, 11.3553, 0.}	{{2.22376, 9.35432, 0.}, {2.86001, 13.5542, 0.}}	{22.3716, 68.4117, 100.}	inf	2850.	4100.54	{3169.09, 5142.79}	51.7902	0.185488	newly	4808.	3800.57	{3300., 4095.}	20.9532	0.103892	Reff	2.02463	2.3474	{2.07929, 2.6384}	20.1929	ar	82 974.	87 476.8	{84 632., 93 092.}	9.93735
46	R0	{2.23409, 9.21289, 0.1}	{2.1659, 9.10138, 0.02}	{{1.44762, 4.00571, 0.}, {2.53075, 11.6597, 0.}}	{20.8616, 37.2104, 116.}	inf	2544.	2707.83	{981.338, 3649.21}	46.8613	0.229792	newly	3017.	3018.78	{2438., 3359.}	11.2131	0.0499063	Reff	2.12542	2.04841	{1.39721, 2.38259}	19.6398	ar	85 163.	77 852.6	{54 440., 89 860.}	15.6385
47	R0	{2.12258, 8.15265, 0.1}	{2.52981, 9.07369, 0.02}	{{1.9862, 6.28417, 0.}, {2.59708, 9.39342, 0.}}	{20.6144, 16.3869, 116.}	inf	2945.	3563.89	{2158.42, 3917.72}	27.558	0.109627	newly	3839.	4702.19	{4057., 5039.}	22.7729	0.0881041	Reff	1.99728	2.37667	{1.90514, 2.44057}	20.0599	ar	82 690.	89 225.8	{79 700., 90 659.}	8.79439
48	R0	{2.71771, 10.4636, 0.1}	{2.19507, 8.06914, 0.01}	{{1.53501, 4.02511, 0.}, {2.9884, 12.8326, 0.}}	{24.7352, 35.5619, 108.}	inf	2061.	1266.99	{575.056, 2021.3}	44.1915	0.297656	newly	2437.	1789.03	{1367., 1987.}	30.7054	0.161476	Reff	2.62862	2.13964	{1.50568, 2.90334}	24.1888	ar	91 716.	78 547.1	{60 068., 93 967.}	15.6941
49	R0	{2.44369, 6.66477, 0.1}	{2.22343, 6.15375, 0.02}	{{1.88643, 4.56746, 0.}, {2.77394, 8.24535, 0.}}	{17.0532, 24.2178, 116.}	inf	1213.	1055.64	{699.22, 1781.3}	34.4038	0.16606	newly	3032.	2510.53	{1543., 3377.}	22.4891	0.124884	Reff	2.39345	2.17912	{1.84961, 2.69984}	16.6806	ar	88 827.	82 947.	{76 043., 92 370.}	8.87415
50	R0	{2.77383, 17.9775, 0.1}	{2.87525, 18.1022, 0.01}	{{2.88523, 18.2127, 0.}, {2.96978, 18.8857, 0.}}	{6.16873, 4.48733, 108.}	inf	14 883.	15 689.2	{15 416.9, 16 708.3}	9.08259	0.0405595	newly	6948.	7355.97	{7213., 7510.}	5.92645	0.0249529	Reff	2.0682	2.11484	{2.12314, 2.1584}	4.30905	ar	92 334.	92 805.8	{93 064., 93 908.}	1.8006
